# RETRACTION: KIF5A Promotes Bladder Cancer Proliferation In Vitro and In Vivo

**DOI:** 10.1155/dim/9767430

**Published:** 2025-06-02

**Authors:** Disease Markers

RETRACTION: D.-W. Tian, Z.-L. Wu, L.-M. Jiang, J. Gao, C.-L. Wu, and H.-L. Hu. “KIF5A Promotes Bladder Cancer Proliferation In Vitro and In Vivo,” *Disease Markers*, no. 2019 (2019). https://doi.org/10.1155/2019/4824902.

The above article, published online on 3 July 2019 in Wiley Online Library (wileyonlinelibrary.com), has been retracted by John Wiley & Sons Ltd.

The retraction has been agreed upon following an investigation into the concerns raised by *Hoya camphorifolia* on PubPeer [1], which identified image manipulation in Figures 1A, 3C, D, 4A, C, and D. Our investigation concluded that the data and the conclusions of the article are considered unreliable.

The authors were made aware of the decision to retract the article but did not provide a response.
